# Left ventricular global longitudinal strain is associated with cardiovascular risk factors and arterial stiffness in chronic kidney disease

**DOI:** 10.1186/s12882-015-0098-1

**Published:** 2015-07-18

**Authors:** Rathika Krishnasamy, Carmel M. Hawley, Tony Stanton, Elaine M. Pascoe, Katrina L. Campbell, Megan Rossi, William Petchey, Ken-Soon Tan, Kassia S. Beetham, Jeff S. Coombes, Rodel Leano, Brian A. Haluska, Nicole M. Isbel

**Affiliations:** Department of Renal Medicine, The University of Queensland at Princess Alexandra Hospital, Brisbane, Australia; School of Medicine, The University of Queensland, Brisbane, Australia; Translational Research Institute, Brisbane, Australia; Cardiovascular Imaging Research Center, The University of Queensland at Princess Alexandra Hospital, Brisbane, Australia; Department of Renal Medicine, Cambridge University Hospital, Cambridge, England; School of Medicine, Griffith University, Brisbane, Australia; School of Human Movement Studies, The University of Queensland, Brisbane, Australia

**Keywords:** Left ventricular function, Global longitudinal strain, Arterial stiffness, Obesity, Uremic toxins

## Abstract

**Background:**

Global longitudinal strain (GLS) has emerged as a superior method for detecting left ventricular (LV) systolic dysfunction compared to ejection fraction (EF) on the basis that it is less operator dependent and more reproducible. The 2-dimensional strain (2DS) method is easily measured and integrated into a standard echocardiogram. This study aimed to determine the relationship between GLS and traditional and chronic kidney disease (CKD)-related risk factors of cardiovascular disease (CVD) in patients with CKD.

**Methods:**

A cross sectional study of patients with moderate CKD stages 3 and 4 (n = 136). Clinical characteristics, anthropometric, biochemical data including markers of inflammation [C-reactive protein (CRP)], uremic toxins [indoxyl sulphate (IS), p-cresyl sulphate (PCS)], and arterial stiffness [pulse wave velocity (PWV)] were measured. Inducible ischemia was detected using exercise stress echocardiogram. GLS was determined from 3 standard apical views using 2-dimensional speckle tracking and EF was measured using Simpson’s rule. Associations between GLS and traditional and CKD-related risk factors were explored using multivariate models.

**Results:**

The study population parameters included: age 59.4 ± 9.8 years, 58 % male, estimated glomerular filtration rate (eGFR) 44.4 ± 10.1 ml/min/1.73 m^2^, GLS −18.3 ± 3.6 % and EF 65.8 % ± 7.8 %. This study demonstrated that GLS correlated with diabetes (r = 0.21, p = 0.01), history of heart failure (r = 0.20, p = 0.01), free IS (r = 0.24, p = 0.005) free PCS (r = 0.23, p = 0.007), body mass index (BMI) (r = 0.28, p < 0.001), and PWV (r = 0.24, p = 0.009). Following adjustment for demographic, baseline co-morbidities and laboratory parameters,GLS was independently associated with free IS, BMI and arterial stiffness (R^2^ for model = 0.30, p < 0.0001).

**Conclusions:**

In the CKD cohort, LV systolic function assessed using GLS was associated with uremic toxins, obesity and arterial stiffness.

**Electronic supplementary material:**

The online version of this article (doi:10.1186/s12882-015-0098-1) contains supplementary material, which is available to authorized users.

## Background

Global longitudinal strain (GLS) has emerged as an objective and more reproducible imaging modality to quantify subtle disturbances in left ventricular (LV) function [1]. GLS detects subendocardial contractility and viability which often precedes an overt impairment of LV function measured by ejection fraction (EF) [2]. It is increasingly reported to be a powerful prognostic tool over other measures of systolic function in various clinical settings including myocardial infarction, cardiomyopathy and valvular heart disease [3–5]. In parallel with these observations, GLS was shown to be a superior predictor of all-cause and cardiovascular (CV) mortality in patients with CKD [6–8].

The pathogenesis of CV disease in CKD is complex and uniquely different resulting in a progressive change in myocardial composition and function [9]. Traditional ‘Framingham’ risk factors such as hypertension, diabetes mellitus, obesity and hypercholesterolemia are highly prevalent in patients with CKD [10] and remain an important component of patient management; however, they do not completely account for the accelerated CV risk in CKD. Renal-specific disturbances known as ‘non traditional risk factors’, including vascular calcification, abnormal bone mineral metabolism (BMM), anemia, hemodynamic overload, inflammation and uremic toxins are putative contributors to cardiac remodeling [11–14]. Similar hemodynamic and metabolic changes also predisposes to arterial stiffening [15]. Whilst arterial and cardiac remodeling can occur in parallel in CKD, arterial stiffness may lead to poor ventricular compliance and hemodynamic decompensation [16, 17].

Early evidence suggests that GLS may have a role in detecting uremic related cardiac remodeling. Kramann et al. reported that strain parameters not only detected LV contractile abnormalities but also correlated with the severity of interstitial myocardial fibrosis and hypertrophy in rat models with uremic cardiomyopathy [8]. There is, however, a lack of information on the association of GLS with CV risk factors in the CKD cohort. Accordingly, the aim of this study was to characterize the association of GLS with traditional and CKD-related risk factors. The current study hypothesized that GLS will be associated with traditional and CKD-related CV risk factors in patients with CKD stage 3 and 4.

## Methods

### Study design

Patients aged 18 or over with an estimated glomerular filtration rate (eGFR) of 25–60 ml/min per 1.73 m^2^, attending the outpatient department of Princess Alexandra Hospital Renal Unit (Brisbane, Australia), were invited to participate. These patients were recruited as part of the open-label randomized controlled trial *L*ongitudinal *A*ssessment of *N*umerous *D*iscrete *M*odifications of *A*therosclerotic *R*isk factors in *K*idney disease (LANDMARK) 3 study, powered for vascular structure and function end-points. The current study included 136 patients who completed the baseline visit and had cardiovascular imaging prior to the intervention of exercise and dietary modification and comprised of 84 % of the overall LANDMARK 3 study population. The study protocol was approved by the Princess Alexandra Human Research Ethics Committee (HREC 2007/190) and was registered at www.anzctr.org.au (Registration Number ANZCTR12608000337370). All of the participants provided written informed consent. Clinical, biochemical and echocardiographic assessments were collected at the time of enrollment for this study.

### Clinical assessment

Demographic data, including an assessment of risk factor status and history of CV disease were recorded; all prescribed and non-prescribed medications were documented. Hypertension and hyperlipidemia were defined by the use of antihypertensive or lipid-lowering therapy, respectively. Diabetes was defined by a history of this diagnosis or use of oral hypoglycemic agents or insulin. During the baseline visit, patients had anthropometric assessment, including height (meters) and weight (kilograms). Body mass index (BMI) was calculated as weight divided by height squared. Overweight and obesity were defined using World Health Organization (WHO) Classification [18]. Previous CV event was defined as a history of documented myocardial infarction, coronary artery bypass surgery, percutaneous coronary intervention, or hospital admission with acute coronary syndrome (ischemic chest pain and/or electrocardiographic [ECG] changes suggestive of ischemia with no elevation in cardiac enzymes), peripheral vascular disease including peripheral revascularization procedure or amputation due to ischemia. Blood pressure (BP) was the average of three seated measurements taken after a 5-minute rest.

### Biochemical assessment

Blood for biochemical analyses was obtained from 10-hour fasting venous samples taken at the baseline visit. Serum concentrations of creatinine, albumin, urate, calcium (corrected for albumin as total calcium − [(Albumin-40)*0.02]), phosphate, parathyroid hormone (PTH), glucose, C-reactive protein (CRP), hemoglobin, and lipids [total cholesterol, low-density lipoproteins (LDL), high-density lipoproteins (HDL) and triglyceride] were determined using standard automated techniques. For this study, eGFR was calculated using the Chronic Kidney Disease- Epidemiology Collaboration (CKD-EPI) equation [19]. Uremic toxins, indoxyl sulphate (IS) and p-cresyl sulphate (PCS), total and free, were measured using the latest ultra- performance liquid chromatography and fluorescence detection method [20].

### Exercise stress echocardiogram and vascular imaging

All patients underwent baseline 2-dimensional transthoracic echocardiography, and six standard views were acquired digitally in a stress protocol. A suitable treadmill protocol for each patient was selected based on Duke Activity Status Index. Patients exercised to maximal capacity, aiming for an age predicted heart rate of >85 %. Echocardiography was repeated immediately post exercise to look for regional abnormalities indicative of inducible ischemia. The resting echocardiographic parameters and stress echo images were analysed off-line side by side by an experienced cardiologist to determine any wall motion abnormalities provoked by stress.

GLS measurements were performed offline using commercially available dedicated automated software (EchoPAC PC, version 11, GE Healthcare, Horten, Norway). Speckles were tracked frame by-frame throughout the LV wall during the cardiac cycle and basal, mid, and apical regions of interest were created. Segments that failed to track were manually adjusted by the operator. GLS was calculated as the mean strain of all 18 segments. Previous studies have demonstrated that healthy individuals have GLS ranging from −16 to −19 % [21, 22]. A cut off at −16 % has been shown to provide important risk stratification and prognostic value [23]. Therefore, in our study we defined impaired GLS as > −16 % (a less negative value reflects a more impaired GLS). Intra- and inter-observer variation for GLS and EF were assessed by intra-class correlation co-efficient (ICC) and compared using Z-scores and Bland Altman plots as published elsewhere [24]. End-diastolic and end-systolic volumes were used to calculate EF by Simpson biplane method from the apical 4- and 2-chamber views [25]. LV mass was calculated with the formula: LV mass = 0.8 × {1.04 [([LV internal dimension + septal wall thickness + posterior wall thickness] ^3^ − LV internal dimension^3^)] + 0.6 g. Left ventricular mass was indexed to height^2.7^, and left ventricular hypertrophy (LVH) was defined as ≥51 g/m^2.7^ for both sexes [26]. Diastolic function was assessed using indices of LV relaxation [E/A; ratio of early (E) and late (A) diastolic transmitral flow velocities and e’; septal mitral annular peak velocities] and LV compliance (E/e’).

Aortic pulse wave velocity (PWV), expressed in meters/second (m/s), was measured using a non-invasive tonometer (SphygomoCor 2000; AtCor Medical, Sydney, Australia) placed over the carotid and femoral arteries at rest. Pressure signals were calibrated using brachial BP and measurements were taken of the distance of the carotid and femoral pulses from a fixed point (the suprasternal notch). The PWV was then calculated using the foot-to-foot method, gated to the cardiac cycle using a 3-lead electrocardiograph [27].

### Statistical analysis

Descriptive statistics were used to represent characteristics at the entry of the study. The data were assessed for normality of distribution and transformed as appropriate. CRP, urine protein-to-creatinine ratio, PTH, PWV, IS and PCS were log transformed. Results were expressed as frequencies and percentages for categorical variables, mean ± standard deviation (SD) for normally distributed variables and median (interquartile range) for non-normally distributed variables. Analysis was carried out by dividing the patients into 2 groups; one with preserved and the other with impaired GLS. Differences between the 2 groups were analysed by chi-square test for categorical data, unpaired *t*-test for continuous normally distributed data and Wilcoxon ranksum test for continuous non-normally distributed data. A sensitivity analysis was done by dividing patients according to above and below the median values of GLS (−18.4 %) to determine whether the observed associations were still robust utilizing a different threshold. The degree of association between GLS as a continuous variable and the variables of interest was assessed using Pearson’s correlation for continuous normally distributed variables and Spearman’s correlation for categorical and non-normally distributed variables. Independent associations with GLS were assessed using stepwise multivariable regression analysis with backward elimination. To further evaluate the relationship between GLS and PWV, a series of linear regression models were constructed using GLS as a dependent variable. The first model included PWV as the sole predictor. Subsequent models were constructed by first adding traditional risk factors as predictors, then CKD-related risk factors, and finally echocardiographic parameters. Multi-colinearity was tested using variable inflation factor measurement. Data were analysed using a standard statistical software program (Stata 13; www.stata.com). P-values less than 0.05 were considered statistically significant for all described analyses.

## Results

### Clinical characteristics

The study included 136 participants (58 % male with a mean age of 59.4 ± 9.8 years and eGFR of 44.4 ± 10.1 mL/min/1.73 m^2^). In this cohort, 67.6 % were obese (BMI > 30 kg/m^2^), 42.7 % had diabetes, 94.9 % had hypertension and 39.7 % had a previous cardiac event. Participants were stratified according to preserved and impaired GLS: preserved GLS ≤ −16 % and impaired GLS > −16 % (a less negative GLS value reflects a more impaired GLS). The association between GLS and clinical characteristics based on cardiac risk factors are shown in Table 1. Participants with impaired GLS had a higher prevalence of diabetes and obesity, higher BMI, uremic toxin (free PCS) and aortic PWV. There was a trend towards an association between inducible cardiac ischemia and impaired GLS (p = 0.05). However, impaired GLS was not associated with age, gender, hypertension, previous CV events or other ‘CKD-related’ risk factors. There were also no differences between the GLS groups in the use of cardiac/anti-hypertensive medications.

**Table 1:** Clinical characteristics of 136 patients according to preserved and impaired GLS

	Preserved GLS	Impaired GLS	p
	(GLS ≤ −16 %)	(GLS > −16 %)	
	N = 106	N = 30	
*Traditional risk factors*			
Age (years)	58.9 ± 10.1	61.1 ± 8.3	0.3
Male (%)	58(55)	21(70)	0.1
Current or previous smoker (%)	70(66)	20(67)	0.9
Diabetes mellitus (%)	38(36)	20(67)	0.003
Fasting Glucose (mmol/L)	6.6 ± 3.0	7.9 ± 3.9	0.04
Hypertension (%)	102(96)	27(90)	0.1
Hypercholesterolemia (%)	70(67)	22(73)	0.7
-Total Cholesterol (mmol/L)	4.5 ± 0.9	4.3 ± 1.4	0.3
-LDL (mmol/L)	2.6 ± 0.9	2.4 ± 1.1	0.4
Previous CV events (%)	40(37.7)	14(46.7)	0.3
History of HF (%)	2(1.9)	3(10)	0.07
Body Mass Index (BMI) (kg/m^2^)	32.4 ± 6.4	35.8 ± 6.2	0.008
Normal: BMI <25	6(5.7)	0(0)	0.03
Overweight: 25 ≤ BMI < 30 (%)	33(31)	5(17)	
Class 1 Obesity: 30 ≤ BMI < 35 (%)	39(37)	9(30)	
Class II and III: Obesity BMI ≥ 35 (%)	28(26)	16(53)	
Blood Pressure (BP)(mmHg)			
Systolic BP	137 ± 19	142 ± 27	0.3
Diastolic BP	81 ± 11	82 ± 14	0.7
Inducible Ischemia on ESE (%)	6(6)	5(17)	0.05
*CKD related risk factors*			
eGFR (ml/min/1.73 m^2^)	44.3 ± 10.1	44.4 ± 10.4	0.9
Urinary protein-to-creatinine ratio (g/mol)	39(11–100)	19(14–88)	0.9
CRP (mg/L)	3.3(1.4-6.8)	4.4(2.4-7.3)	0.2
Albumin (g/L)	37.6 ± 3.6	37.4 ± 4.7	0.8
Urate (mmol/L)	0.46 ± 0.1	0.45 ± 0.1	0.6
Hemoglobin (g/L)	132 ± 15	131 ± 16	0.9
Corrected calcium (mmol/L)	2.35 ± 0.1	2.33 ± 0.1	0.3
Phosphate (mmol/L)	1.11 ± 0.17	1.15 ± 0.16	0.2
PTH (pmol/L)	8(6–13)	12(7–16)	0.2
Free indoxyl sulphate (μmol/L)	0.31(0.22-0.50)	0.37(0.30-0.51)	0.07
Free P-cresyl sulphate (μmol/L)	1.38(0.71-2.14)	1.90(1.43-2.61)	0.01
Pulse wave velocity (m/s)	9.0(7.3-10.7)	10.4(9.1-13)	0.03
*Medication*			
ACEi/ARB (%)	88(84.6)	26(89.7)	0.5
Βeta blockers (%)	41(39.4)	11(37.9)	0.9
Calcium channel blockers (%)	49(47)	15(51.7)	0.7
Diuretics (%)	39(38.7)	14(48.3)	0.4

Data are mean ± standard deviation, median (interquartile range) or number (%)

GLS; global longitudinal strain, LDL; low density lipoprotein, CV; cardiovascular, HF; heart failure, CKD; chronic kidney disease, eGFR; estimated glomerular filtration rate, CRP; C-reactive protein, PTH; parathyroid hormone, ACEi; angiotensin converting enzyme inhibitor, ARB; angiotensin receptor blocker, ESE; exercise stress echocardiogram

### Relationship between GLS and with indices of LV structure and function

Mean EF was 65.8 ± 7.8 % and mean GLS was −18.3 ± 3.6 %. Notably, 49 % of participants had LVH, primarily with concentric hypertrophy. Table 2 presents the association of GLS and echocardiographic parameters. Impaired GLS was associated with lower EF, higher left ventricular mass index (LVMI), higher left ventricular end systolic volume (LVESV), poorer left ventricular relaxation and compliance (assessed using e’ and E/e’). There was no association between GLS and left atrial (LA) volume or LV geometry.

**Table 2:** Echocardiographic characteristic according to impaired and preserved GLS

	Preserved GLS	Impaired GLS	p
	(GLS ≤ −16 %)	(GLS > −16 %)	
	N = 106	N = 30	
Ejection fraction (%)	67.6 ± 6.9	59.5 ± 7.5	<0.001
LVESV (ml)	25.7 ± 11.7	33.2 ± 20.8	0.01
LVEDV (ml)	75.4 ± 23.9	80.1 ± 33.6	0.4
LVESD (mm)	2.81 ± 0.53	3.01 ± 0.83	0.1
LVEDD (mm)	4.77 ± 0.58	4.79 ± 0.87	0.9
LVMI (g/m^2.7^)	50.3 ± 11.7	56.3 ± 20.7	0.04
RWT	0.49 ± 0.12	0.51 ± 0.11	0.3
LVH (LVMI ≥ 51 g/m^2.7^)	53(50)	16(53)	0.8
- eccentric LVH	8(15)	2(13)	
- concentric LVH	45(85)	14(87)	
LV compliance			
- E/e’	12.3 ± 4.2	15.5 ± 10.9	0.02
LV relaxation			
- E/A	1.0 ± 0.4	0.9 ± 0.3	0.2
- e’ (cm/s)	0.06 ± 0.01	0.05 ± 0.01	0.007
LA volume (ml)	61.5 ± 19.4	62.0 ± 23.2	0.9

GLS; global longitudinal strain, LVESV; left ventricular end systolic volume, LVEDV; left ventricular end diastolic volume, LVESD; left ventricular end systolic diameter, LVEDD; left ventricular end diastolic diameter, LVMI; left ventricular mass index, RWT; relative wall thickness, LVH; left ventricular hypertrophy, LA; left atrial

### Traditional and CKD-related risk factors as predictors of GLS

**Fig. 1:**
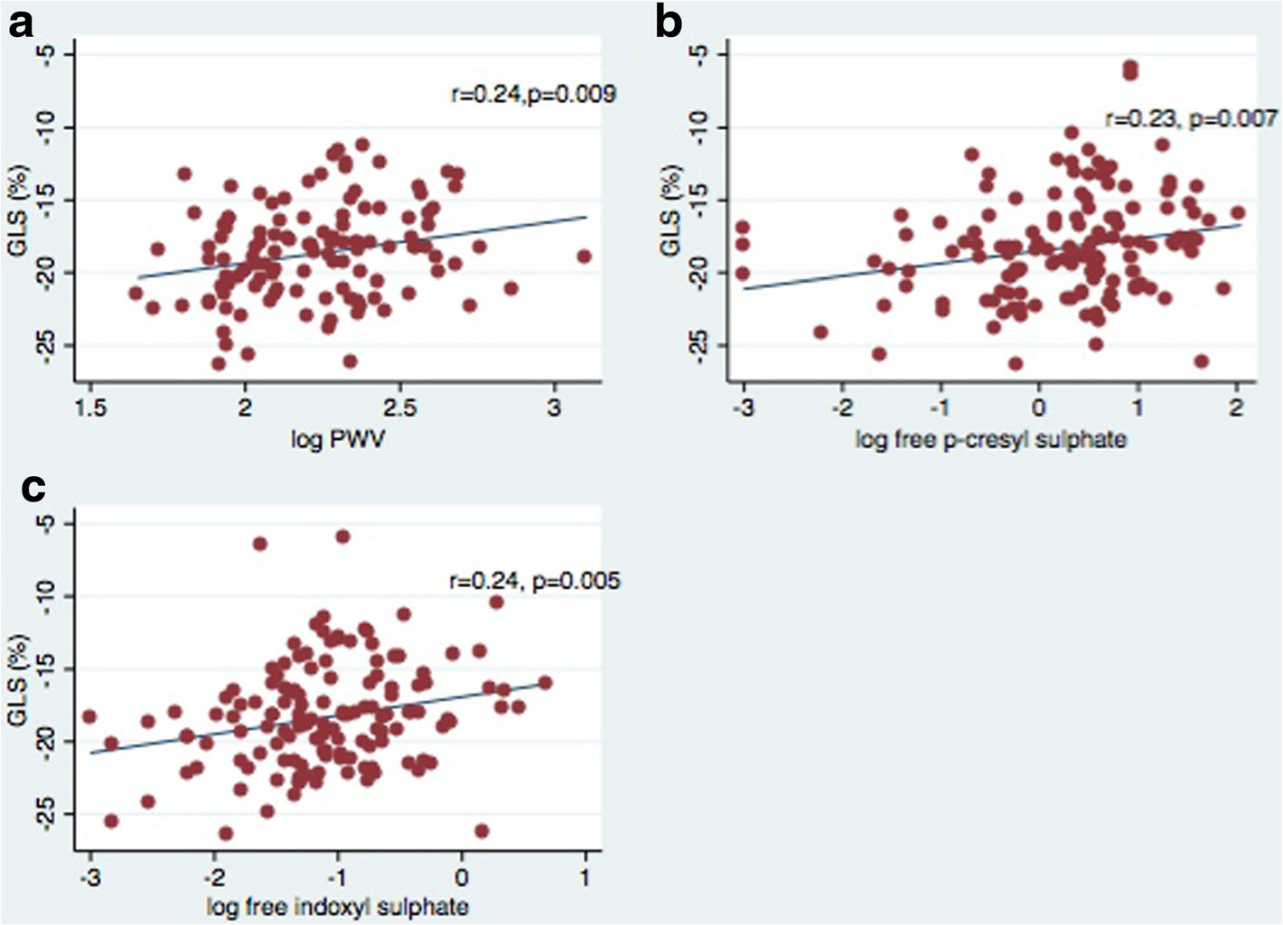
**a-c**: The association between global longitudinal strain (GLS) and **a** aortic pulse wave velocity (PWV), **b** free p-cresyl sulphate and **c** free indoxyl sulphate

Table 3 represents the bivariate and adjusted associations between GLS and relevant CV risk factors. In bivariate analysis, GLS correlated with several important traditional risk factors including history of diabetes, heart failure (HF) and BMI. In addition, GLS also correlated with CKD-related risk factors (Fig. 1a-c) including free IS (r = 0.24, p = 0.005), free PCS (r = 0.23, p = 0.007) and PWV (r = 0.24, p = 0.009). Using stepwise linear regression adjusting for demographic, traditional and CKD-related risk factors, GLS remained independently associated with aortic PWV, free IS and BMI (R^2^ for model = 0.30, p < 0.0001).

**Table 3:** Bivariate and Multivariate association with Global Longitudinal Strain

Variable	Bivariate Analysis		Multivariate Model (R^2^ = 0.30, n = 114 p < 0.0001)	
	r	p	^#^Â coefficient (95 % CI)	p
*Traditional risk factors*				
Age (years)	0.01	0.9	−0.06(−0.13,0.01)	0.1
Gender (Male)	0.15	0.09	0.92(−0.23,2.07)	0.1
Smoking history	0.0004	0.99		
Hypertension	−0.09	0.3		
Diabetes	0.21	0.01		
Hypercholesterolemia	0.06	0.47		
Previous CV events	0.07	0.43		
HF	0.2	0.01		
BMI (kg/m^2^)	0.28	<0.001	0.1(0.02,0.2)	0.02
Peripheral systolic BP (mmHg)	0.08	0.3	−0.28(−0.07,0.01)	0.1
Peripheral diastolic BP (mmHg)	0.14	0.1	0.06(−0.002, −0.13)	0.06
Fasting glucose (mmol/L)	0.1	0.2		
Inducible ischemia on ESE (%)	0.14	0.1	2.05(−0.03,4.12)	0.05
*CKD- related risk factors*				
eGFR (ml/min/1.73 m^2^)	0.04	0.6		
Urine PCR (g/mol)^	0.08	0.4		
CRP (mg/L)^	0.16	0.07		
Albumin (g/L)	0.01	0.9		
Calcium (mmol/L)	−0.13	0.2	−4.8(−9.6,0.06)	0.05
Phosphate (mmol/L)	0.09	0.3		
PTH (pmol/L)^	0.1	0.3		
Urate (mmol/L)	−0.1	0.9		
Hemoglobin (g/L)	0.03	0.7		
Free ICS (μmol/L)^	0.24	0.005	0.9(0.07,1.68)	0.03
Free PCS (μmol/L)^	0.23	0.007		
PWV (m/s)^	0.24	0.009	3.29(0.53,6.03)	0.02

CI; confidence interval, HF; heart failure, BMI; body mass index, BP; blood pressure; CKD; chronic kidney disease, eGFR; estimated glomerular filtration rate, ESE; exercise stress echocardiogram,  PCR; protein-to-creatinine ratio, CRP; C-reactive protein, PTH; parathyroid hormone, ICS; indoxyl sulphate, PCS; p-cresyl sulphate; PWV pulse wave velocity

^log transformed

^#^The coefficient notes the per unit change in GLS

### Left ventricular –arterial association

To further distinguish the association between GLS and arterial stiffness, we compared regression and squared correlation coefficients for these parameters adjusting for traditional risk factors, CKD-related risk factors and echocardiographic parameters (Table 4). There was a statistically significant association between GLS and aortic PWV, which remained following adjustment for relevant traditional risk factors (Model 2: age, gender, diabetes, systolic BP, diastolic BP, HF, BMI, inducible ischemia) and CKD-related risk factors (Model 3: addition of eGFR, CRP, corrected calcium and uremic toxins to Model 2). The independent association between GLS and PWV also persisted following adjustment for relevant echocardiographic parameters (Model 4: addition of EF, LVMI and E/e’ to Model 3) with a decrease in the correlation coefficient.

**Table 4:** Multivariate regression models for GLS: assessing the independent contribution of aortic stiffness

	Model R^2^	^#^Â coefficient (95 % CI)	p
1: Unadjusted PWV	0.06	2.9(0.72,5.04)	0.009
2: 1+ traditional risk factors*	0.26	3.2(0.57,5.81)	0.01
3: 2+ CKD-related risk factors**	0.30	3.34(0.54,6.15)	0.02
4: 3+ echo parameters***	0.44	2.3(0.48,4.06)	0.01

*Traditional risk factors include age, gender, diabetes, heart failure, BMI, systolic BP, diastolic BP, inducible ischemia

**CKD related risk factors include eGFR, CRP, free IS, free PCS, corrected calcium

***Echocardiogram parameters: EF, E/e’, LVMI

^#^The coefficient notes the per unit change in GLS, PWV; pulse wave velocity, CKD; chronic kidney disease, CI; confidence interval

### Sensitivity analysis

The associations between GLS, clinical characteristics and indices of LV structure and function were repeated with a GLS cut –off at median value of −18.4 % (Additional file 1: Table S1A and S2A). Participants with lower GLS (> − 18.4 %) were still found to have higher BMI, uremic toxins (free IS and free PCS) and aortic PWV.

## Discussion

This study showed in patients with established CKD stage 3 and 4, LV systolic function assessed by GLS was independently associated with aortic PWV, uremic toxins and BMI. Other traditional risk factors did not demonstrate an association with GLS. Importantly, to our knowledge this is the first study to identify the highly significant association between aortic stiffness and GLS.

There are few studies that have assessed clinical factors associated with GLS in the general population. The current study explored the associations between risk factors and GLS in CKD. In a meta-analysis, Yingchoncharoen et al. found systolic BP was an important source of variation in GLS values [28]. Dalen et al. have also reported that among healthy individuals increasing age and male gender were associated with worse GLS [29]. The current findings did not show significant associations between GLS and SBP, age or gender. There are several possible explanations for these differences including our study cohort consisted of CKD patients that have many distinctive characteristics compared to the general population. A history of hypertension was universal and BP was well controlled in this study cohort. In addition, historical values of BP readings were not available for comparison and the relatively small sample size could account for the lack of association between GLS and BP seen in this study.

CKD is a unique risk factor for cardiac remodeling; studies have demonstrated that this occurs early and is significantly worse in CKD patients compared to non-CKD [30, 31]. The structural changes are characterized by cardiomyocyte cell loss and hypertrophy, increased wall stress, dilatation or thinning of ventricular wall, scar formation and myocardial fibrosis which progresses to a maladaptive response and results in functional decompensation [32, 33]. Previous work has also demonstrated that hemodynamic and metabolic changes associated with the uremic milieu can result in endothelial dysfunction and a cascade of vascular injury in this cohort [34–36]. Endothelial dysfunction is a major pathogenic mechanism for exaggerated atherosclerosis and arteriosclerosis resulting in reduced vascular and myocardial compliance, increased vascular calcification and stiffening [37]. Arterial stiffness, assessed using PWV, has been widely described and is associated with adverse CV outcome in CKD [38, 39]. It has been hypothesized that arterial stiffness may have deleterious effects on LV filling pressure resulting in greater LV wall stress and stiffness and subsequent injury to the subendocardium which is highly sensitive to wall stress and myocardial oxygen demand [40]. As GLS quantifies longitudinal contraction, especially in the subendocardial fibers, it may be a more sensitive marker of systolic dysfunction occurring prior to overt clinical disease [2, 41]. In this study, the independent association of arterial stiffness and GLS persisted following adjustment for inducible ischemia, LVMI, EF and indices of diastolic function. Whilst classically vascular stiffness is directly related to ventricular stiffness and diastolic function, our study indicates that LV systolic function can be compromised in response to ventricular- vascular stiffening in patients with moderate CKD.

The present study also demonstrated that increasing BMI was associated with worsening GLS in patients with moderate CKD. Obesity is an established risk factor for cardiomyopathy and is a growing problem in CKD. Obesity results in various metabolic and neuro-humoral alterations that can augment myocardial remodeling. Excessive free fatty acids through alteration of fatty acid β-oxidation rates has been shown to increase myocardial oxygen consumption and impair myocardial contractility [42]. Obesity is related to activation of inflammatory cytokines, especially tumour necrosis factor (TNF), that contribute to fibrotic changes of the myocardium [43]. Activation of the sympathetic and renin-aldosterone system is also widely demonstrated in obese persons and can further facilitate cardiac damage [44]. Some of these factors co-exist or are attenuated in CKD patients [45]. As a result, there are numerous maladaptive changes of the myocardium that overlap between CKD and obesity, including abnormal LV relaxation, hypertrophy and interstitial fibrosis [46].

CKD- related risk factors are increasingly thought to amplify the multifaceted mechanisms of cardiovascular disease. Accordingly, this study showed a novel and independent association between the free circulating concentrations of uremic toxin IS and worsening GLS in this cohort with moderate CKD. Protein bound uremic toxins, such as IS and PCS, have been shown to accumulate with progression of CKD and are associated with adverse CV outcomes [47]. IS and PCS are both by-product of bacterial protein fermentation in the large bowel and are not efficiently cleared in the presence of kidney disease. Although these toxins are primarily protein-bound, their free fraction, which is the unbound metabolically active component, increases with deterioration of kidney function [48]. Further, these toxins have been closely linked to the synthesis of inflammatory mediators and up-regulation of inflammation among CKD patients [49, 50]. *In vitro* studies demonstrate exposure to free IS and PCS results in activation of the Nuclear Factor-kappa B (NF-kB) pathway [49] and exposure to IS in particular stimulates mitogen-activated protein kinase (MAPK) pathways, with subsequent fibrotic, oxidative and pro-inflammatory effects on the myocardium [14].

Myocardial ischemia is a pivotal factor for cardiac remodeling in CKD and GLS was previously reported to provide prognostic information on myocardial ischemia and infarct size [51]. This study observed a trend towards an association between GLS and myocardial ischemia (p = 0.05).

This investigation is a comprehensive analysis of CV risk factors and GLS. However, a cause-effect relationship was unable to be identified due to the cross-sectional nature of the study. In this study traditional risk factors were well controlled which may have limited our ability to detect associations between these parameters and GLS. Moreover, the study was limited to subjects with an eGFR of 25 – 60 ml/min/1.73 m^2^ and included only 30 patients with impaired GLS. Even though a large number of patient characteristics were adjusted for, the possibility of residual confounding cannot be excluded. Larger studies are required to further explore associations with GLS in CKD.

## Conclusions

This study demonstrated the associations of multiple traditional and CKD-related risk factors with LV systolic function assessed using GLS in patients with CKD. LV systolic function assessed using GLS was associated with uremic toxins, obesity and arterial stiffness. Future studies are required to assess whether therapeutic strategies to modify these CV risk factors can result in improved LV function.

## Additional file

Additional file 1: Table S1. A: Clinical characteristics of 136 patients according to GLS values above and below the median. **Table S2.** A: Echocardiographic characteristic according values above and below the median.

## Abbreviations

GLS: Global longitudinal strain
LV: Left ventricular
EF: Ejection fraction
2-DS: 2-dimensional strain
CKD: Chronic kidney disease
CRP: C-reactive protein
IS: Indoxyl sulphate
PCS: P-cresyl sulphate
PWV: Pulse wave velocity
eGFR: Glomerular filtration rate
BMI: Body mass index
CVD: Cardiovascular disease
CV: Cardiovascular
BMM: Bone mineral metabolism
WHO: World Health Organization
ECG: Electrocardiographic
BP: Blood pressure
LDL: Low-density lipoproteins
HDL: High-density lipoproteins
PTH: Parathyroid hormone
CKD-EPI: Chronic Kidney Disease- Epidemiology Collaboration
LVH: Left ventricular hypertrophy
LVMI: Left ventricular mass index
LVESV: Left ventricular end systolic volume
HF: Heart failure
TNF: Tumour necrosis factor
NF-kB: Nuclear Factor-kappa B
MAPK: Mitogen-activated protein kinase
