# Supplementary material for: Left ventricular global longitudinal strain is associated with cardiovascular risk factors and arterial stiffness in chronic kidney disease
Source: BMC Nephrol. 2015 Jul 18;16:106. doi: 10.1186/s12882-015-0098-1 (PMC4506621; doi:10.1186/s12882-015-0098-1)
Supplement: Additional file 1: Table S1. — A: Clinical characteristics of 136 patients according to GLS values above and below the median. Table S2. A: Echocardiographic characteristic according values above and below the median. [file 12882_2015_98_MOESM1_ESM.docx]

Supplemental Table 1 A: Clinical characteristics of 136 patients according to GLS values above and below the median

|  | GLS≤-18.4%  N = 66 | GLS>-18.4%  N = 70 | p value |
| --- | --- | --- | --- |
| *Traditional risk factors* | | | |
| Age (years) | 59.8±10.2 | 59.0±9.4 | 0.6 |
| Male (%) | 34(51.5) | 45(64.2) | 0.09 |
| Current or previous smoker (%) | 44(66.7) | 46(65.7) | 0.9 |
| Diabetes mellitus (%)  Fasting Glucose (mmol/L) | 25(37.7)  6.6±3.2 | 36(51.4)  7.1±3.3 | 0.1  0.3 |
| Hypertension (%) | 66(100) | 63(90) | 0.01 |
| Hypercholesterolemia (%)  -Total Cholesterol (mmol/L)  -LDL (mmol/L) | 42(64.6)  4.5±0.9  2.6±0.9 | 50(71.0)  4.4±1.2  2.5±1.1 | 0.09  0.6  0.5 |
| Previous CV events (%) | 24(36.4) | 30(42.9) | 0.7 |
| History of HF (%) | 1(1.5) | 4(6.0) | 0.2 |
| Body Mass Index (kg/m^2^)  Normal: BMI <25  Overweight: 25≤BMI<30(%)  Class 1 Obesity: 30≤BMI <35(%)  Class II and III: Obesity BMI≥ 35(%) | 31.4±5.6  5(7.6)  23(34.8)  24(36.3)  14(21.2) | 34.8±6.8  1(1.4)  15(21.4)  24(34.3)  30(42.9) | 0.002  0.02 |
| Blood Pressure (BP)(mmHg)  Systolic BP  Diastolic BP | 137±20  79±11 | 138±22  83±14 | 0.8  0.06 |
| Inducible Ischemia on ESE (%) | 4(6.1) | 7(10.0) | 0.5 |
| *CKD related risk factors* | | | |
| eGFR (ml/min/1.73m^2^) | 44.3±9.2 | 44.7±10.9 | 0.7 |
| Urinary protein-to- creatinine ratio (g/mol) | 45(11-91) | 30(12-100) | 0.4 |
| CRP (mg/L) | 3.1(1.6-6.2) | 3.9(1.8-7.6) | 0.2 |
| Albumin (g/L) | 37.6±3.6 | 37.4±4.3 | 0.9 |
| Urate (mmol/L) | 0.45±0.1 | 0.46±0.1 | 0.9 |
| Hemoglobin (g/L) | 132±15 | 131±16 | 0.6 |
| Corrected calcium (mmol/L) | 2.36±0.1 | 2.33±0.1 | 0.05 |
| Phosphate (mmol/L) | 1.11±0.16 | 1.12±0.19 | 0.7 |
| PTH (pmol/L) | 8(6-12) | 10(7-16) | 0.1 |
| Free indoxyl sulphate (μmol/L) | 0.30(0.21-0.45) | 0.37(0.24-0.58) | 0.01 |
| Free P-cresyl sulphate (μmol/L) | 1.38(0.71-2.14) | 1.76(1.19-2.81) | 0.02 |
| Pulse wave velocity (m/s) | 8.3(7.1-10.7) | 9.9(8.1-11.5) | 0.02 |
| *Medication* | | | |
| ACEi/ARB (%) | 57(87.7) | 26(82.4) | 0.5 |
| Βeta Blockers (%) | 24(36.9) | 28(41.2) | 0.7 |
| Calcium channel blockers(%) | 36(55.4) | 30(44.1) | 0.2 |
| Diuretics (%) | 24(36.9) | 30(44.1) | 0.4 |

Data are mean ± SD, median (interquartile range) or number (%)

GLS; global longitudinal strain, LDL; low density lipoprotein, CV; cardiovascular, HF; heart failure, CKD; chronic kidney disease, eGFR; estimated glomerular filtration rate, CRP; C-reactive protein, PTH; parathyroid hormone, ACEi; angiotensin converting enzyme inhibitor, ARB; angiotensin receptor blocker, ESE; exercise stress echocardiogram

Supplemental Table 2 A: Echocardiographic characteristic according values above and below the median

|  | GLS≥-18.4%  N = 66 | GLS<-18.4%  N = 70 | p value |
| --- | --- | --- | --- |
| Ejection Fraction (%) | 69.0±6.8 | 62.7±7.3 | <0.001 |
| LVESV (ml) | 24.7±11.8 | 30.3±16.2 | 0.02 |
| LVEDV (ml) | 73.7±23.7 | 79.1±28.5 | 0.3 |
| LVESD (mm) | 2.75±0.55 | 2.96±0.65 | 0.06 |
| LVEDD (mm) | 4.72±0.62 | 4.83±0.67 | 0.3 |
| LVMI (g/m^2.7^) | 49.6±12.1 | 53.5±0.1 | 0.1 |
| RWT | 0.49±0.12 | 0.50±0.11 | 0.5 |
| LVH (LVMI≥51 g/m^2.7^)   - eccentric LVH - concentric LVH | 30(45.6)  5(16.7)  25(83.3) | 39(51.5)  5(12.8)  34(87.2) | 0.3 |
| LV compliance   - E/e’ | 12.3±4.2 | 13.6±7.8 | 0.3 |
| LV relaxation   - E/A - e’(cm/s) | 1.0±0.4  0.06±0.01 | 0.9±0.3  0.05±0.01 | 0.08  0.005 |
| LA volume (ml) | 59.9±20.4 | 63.3±20.2 | 0.3 |

LVESV; left ventricular end systolic volume, LVEDV; left ventricular end diastolic volume, LVESD; left ventricular end systolic diameter, LVEDD; left ventricular end diastolic diameter, LVMI; left ventricular mass index, RWT; relative wall thickness, LVH; left ventricular hypertrophy
